# Supplementary figures and images for: The highly dynamic pangenome of basal chordates is enriched in defence and immunity genes and is inherited following the Mendelian law
Source: PLoS Genet. 2025 Aug 18;21(8):e1011833. doi: 10.1371/journal.pgen.1011833 (PMC12373286; doi:10.1371/journal.pgen.1011833)

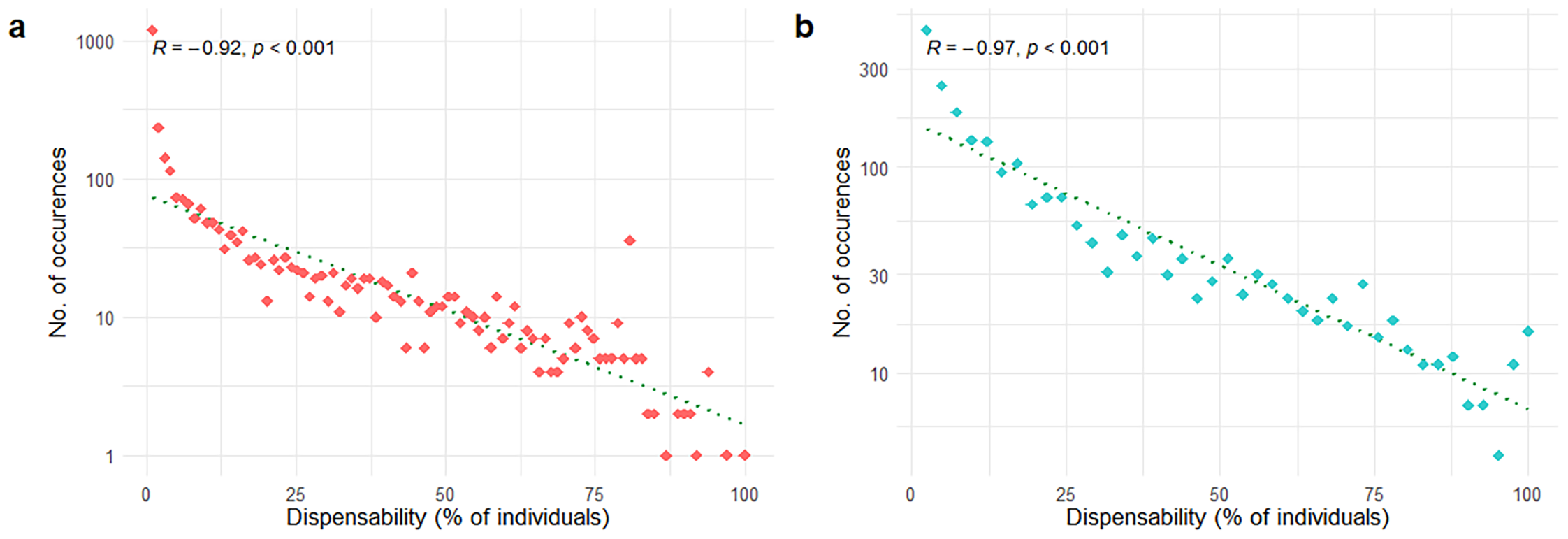

Supplement: S1 Fig — (TIFF) [file pgen.1011833.s006.tiff]

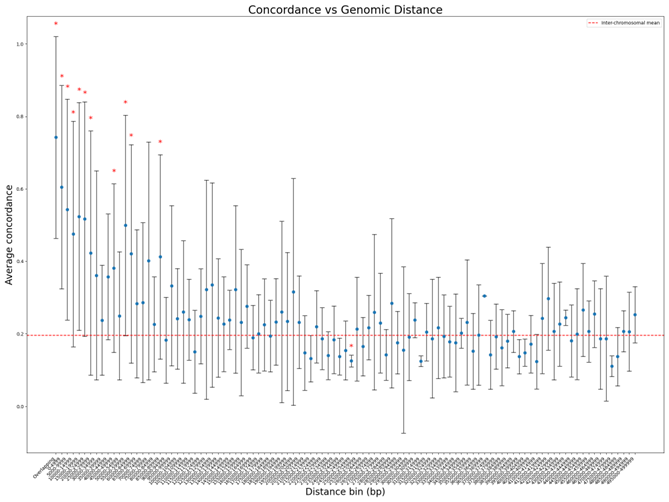

Supplement: S2 Fig — Statistically significant differences (Wilcoxon rank-sum tests, FDR-corrected p-value < 0.05) compared with inter-chromosomal dispensable gene pairs (dashed red line) are highlighted by an asterisk. Note that the genes included in the integrated BelcheriHV-1 were omitted. (TIF) [file pgen.1011833.s007.tif]

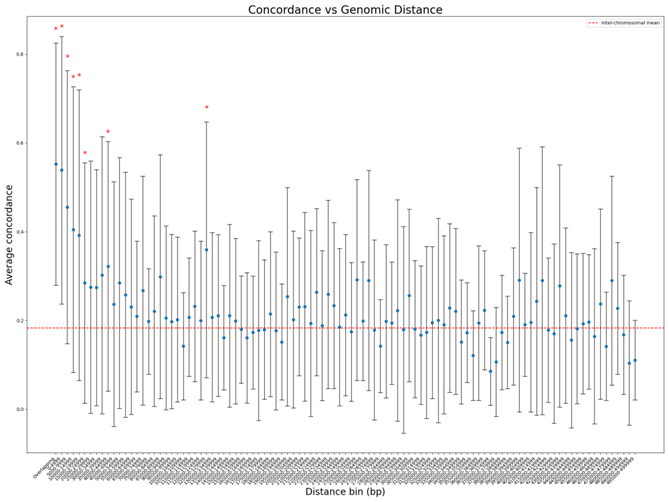

Supplement: S3 Fig — Statistically significant differences (Wilcoxon rank-sum tests, FDR-corrected p-value < 0.05) compared with inter-chromosomal dispensable gene pairs (dashed red line) are highlighted by an asterisk. (TIF) [file pgen.1011833.s008.tif]

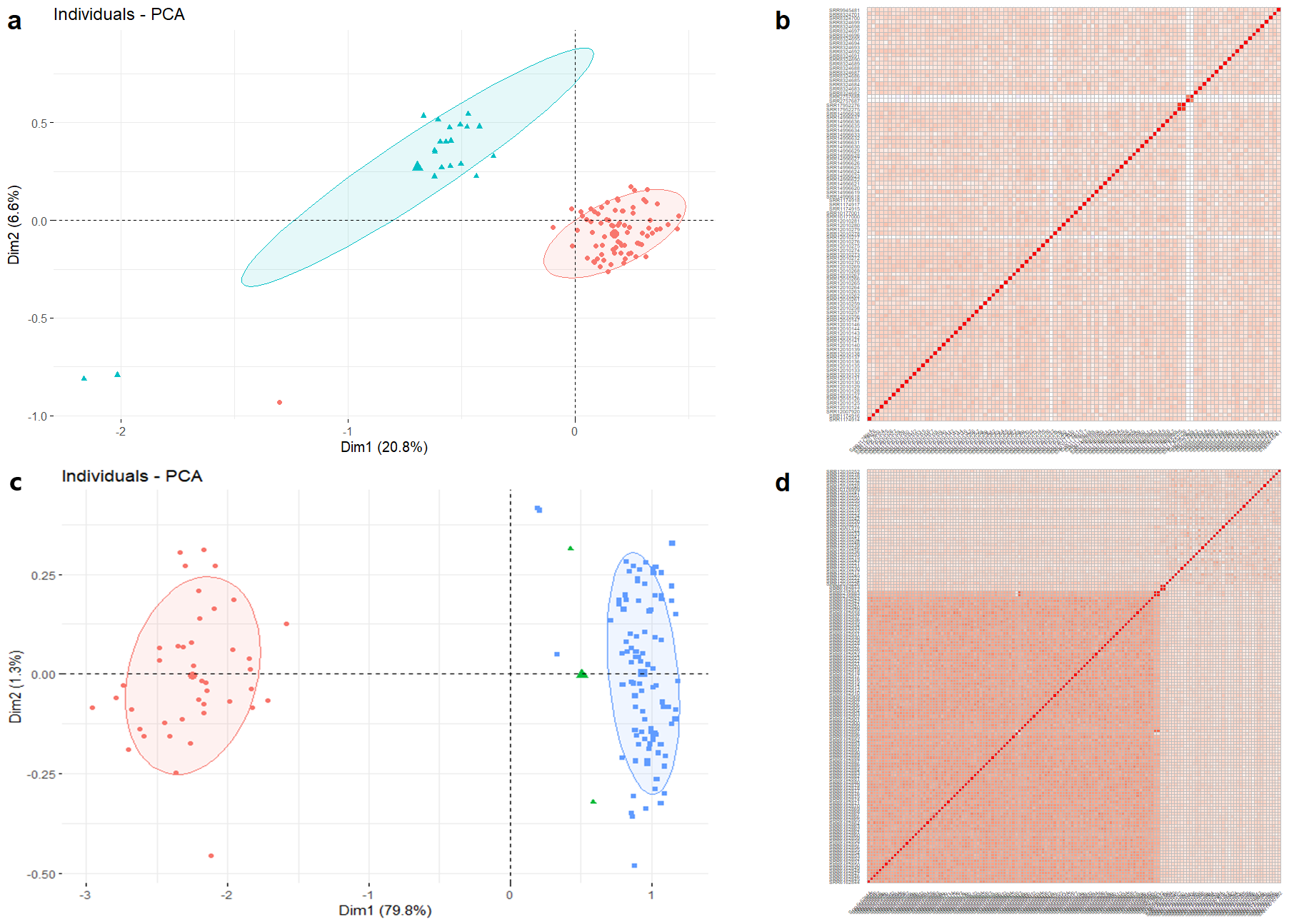

Supplement: S4 Fig — The B. belcheri samples in the PCA are coloured according to the presence of the integrated BelcheriHV-1 genome (YES: blue, NO: red). Instead, the B. floridae samples in the PCA are coloured by experiment, with the blue ones referring to the parental + offspring dataset. (TIF) [file pgen.1011833.s009.tif]

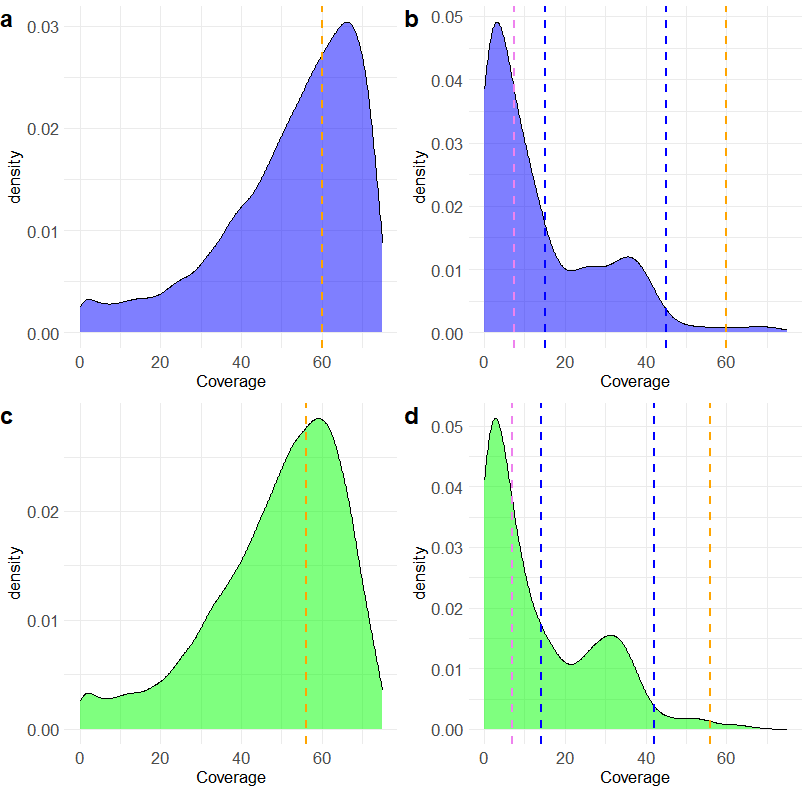

Supplement: S5 Fig — The orange dotted lines indicated the average coverage computed based on core genes. Gene coverage plots for the dispensable genes in the female (b) and male (d) genotypes. The pink dotted lines indicate the cut-off used to determine the absence of a given gene, whereas the two blue lines indicate the range used to determine the genes in hemizygosity, and the orange lines refer to the expected coverages of core genes. (TIFF) [file pgen.1011833.s010.tiff]
