# Supplementary material for: The highly dynamic pangenome of basal chordates is enriched in defence and immunity genes and is inherited following the Mendelian law
Source: PLoS Genet. 2025 Aug 18;21(8):e1011833. doi: 10.1371/journal.pgen.1011833 (PMC12373286; doi:10.1371/journal.pgen.1011833)
Supplement: Attachment — Submitted filename: response_to_reviewers.pdf [file pgen.1011833.s016.pdf]

Padova, May 23, 2025

Dear PLOS Genetics Editor and Reviewers,

We thank you for your effort in revising our paper, providing us with several constructive suggestions that were welcomed and used to improve the overall narrative and results' clarity. Below, we provided a point-by-point response letter to each comment.

On behalf of my co-authors,

Umberto Rosani

**Reviewer #1**

This manuscript presents a study of the gene repertoire in the amphioxus species B. belcheri and B. floridae. They use publicly available genomic and transcriptomic data from a large number of B. belcheri and B. floridae individuals. They study presence-absence variation (PAV) and non-reference sequences (NRS) in a mainly gene-centric approach to gain insight into the genes that are dispensable in these species.
Amphioxus species are known to have an extremely high level of heterozygosity, and thus studying genomic diversity in an alternative way by studying the genes that are dispensable may shed light on this extreme case of genomic diversity at the base of the chordate tree. However, the present manuscript does not fulfil the expectations expressed in the title and abstract, nor does it meet proper scientific publishing standards in its current form. A detailed revision of the manuscript in its current form cannot be done properly. Several important issues would need to be addressed beforehand.

First, the text is long and complicated. It is difficult to follow and lacks rationality and structure in many places. The whole manuscript needs a thorough rewrite with the aim of making it more concise and understandable to an expert in the field. Please take the time to improve the text, give each paragraph a message and link them to an overall rationale.

We apologize for the excessive use of technical language, which likely reduced the overall effectiveness of the original narrative. In response to this observation, we have substantially reorganized the text to ensure a logical flow of information and to facilitate the reader's understanding of the topics. We have rewritten several sections to reduce bioinformatics jargon, simplify overly complex paragraphs, and more clearly introduce the analyses and their underlying rationale.

Second, the results are a compendium of figures and statistics without a clear rationale or link between them. In other words, they lack logic and structure. In addition, the text does not really address all the results shown in the figures, and it is not clear which species or test is being referred to at each point. Figure labels and captions do not always stand alone. Please ensure that the main figures show the reader what they need to see to understand the results and the rationale of the manuscript. Make sure that the figures and their captions are self-explanatory. Consider adding a schematic figure explaining the methods and classification of regions/genes as core, shell/cloud or dispensable.

We appreciate the reviewer's valuable feedback regarding the clarity and structure of the results and figures. In response, we have thoroughly revised the results section to establish a clearer logical flow between figures and analyses, explicitly stating the rationale behind each set of results. We have simplified some figures, moving some panels to the supplementary material for the sake of clarity, and carefully rewritten figure legends to ensure they are self-explanatory and provide sufficient context for standalone interpretation. We believe that in the current version the different sections of the results are better linked, and the overall rationale is much easier to follow compared with the original draft.

Third, the term "pangenome" is not used in the same way in all studies (see Matthews CA et al., A gentle introduction to pangenomics. Brief Bioinform. 2024); its use has evolved since it was first used in the early 2000s with the advent of new sequencing technologies and the availability of data. In this study, the authors use a classic gene-centered approach rather than a fully de-novo assembly approach, although they do re-assemble the unmapped genes to include non-reference genes. I agree that this is a valid and informative approach given the available data, but I would like to see all this clarified and discussed in the manuscript.

Thanks for this point. We have now included a clarification of the classical and modern pangenome concepts in the introduction. We modified the explanation of pangenome, shell, cloud and dispensable genes for the sake of clarity. In the result section, we clearly stated our aim, the rationale behind the choice of using a gene-centric approach, and the limitations associated with this methodology and the availability of whole genome resequencing data based on short reads only.

Fourth, at several points in the text, the authors seem to argue that it is not universal for all species to have a pangenome, but that some species might not have one (see, for example, the first subtitle of results "PAV analysis reveals the existence of a pangenome in two lancelet species"). Please develop how a given species might not have a pangenome.

It wasn't our intention to state that some animals do not possess a pangenome. We mostly argued that the pangenome composition and extension is different between taxa. We corrected the sentences that might be miss-interpretated, including the title at line 110 (now: "Lancelet genomes are characterized by widespread PAV").

Finally, as a bioinformatic work, I would like to see all the code used to get the results in a public repository, such as GitHub.

Regarding this aspect, we aim to note that we have already referred to publicly available pipelines for the PAV analysis (lines 569 and 586 for the part related to PAV analysis and GO/Pfam enrichment). The subsequent analyses and rendering of tabular data (essentially matrices of presence-absence and coverage and expression levels per gene) have been performed using R packages (as stated at line 607-608).

**Reviewer #2**

This paper by Rosani et al. describes the assembly and analysis of the pangenomes of two closely related cephalochordates, Branchiostoma floridae and belcheri. The starting point are the publicly available genome assemblies from both species and genomic read libraries from a large number of individuals of each species. Using their own presence-absence variation (PAV) analysis pipeline the author then identified hundreds (!) of genes that are either present or absent in individuals of each population for both species. This is then followed by classifying them based on common features of the encoded protein including gene ontology and domain content. Intriguingly, but may be not surprisingly, the main commonality that emerges (consistently in both species) is that they appear to encode genes with predicted immune function. This is in line with the widely held view that variations of immune genes within a population are important features to allow for the survival of species in light of the rapidly evolving microbial universe from which new pathogens can easily arise. The author here now demonstrate clearly that it is not just SNPs but also the PAVs that contribute to this intraspecies genetic diversity. This manuscript is of high interest for a very broad range of scientists including but not limited to those who study structural features of genomes and comparative immunologists. It is a very important piece of work that will gather significant attention.

1 ) My main concern is that the beginning of results section uses too much bioinformatics jargon and is too densely written such that it is hard and tedious for the broad audience to keep the attention until they later on can identify with domain and gene names that are familiar. I recommend recruiting the help of a wet lab molecular biology colleague to revise the text such that it becomes more easily accessible – it will be worth it.

Thank you for pointing this out. We have modified the parts where we have used technical phrasing and bioinformatic jargon to improve the readability of the content by general readers and tried to improve the narrative.

2 ) After reading this manuscript many immunologist will likely wonder about whether the Branchiostoma RAG1/2-like genes (thought to a the ancestor of the V(D)J recombinase) are among the genes with PAV or not. A short note on this would be good to include.

We thank the reviewer for this interesting suggestion, which is directly relevant and in line with our other results showing the significant involvement of amphioxus immune-related genes in PAV. Based on the reports by Dong et al. 2005 (https://doi.org/10.1016/j.fsi.2004.11.001), Zhang et al. 2013 (https://doi.org/10.1073/pnas.1318843111) and Huang et al., 2016 (https://doi.org/10.1016/j.cell.2016.05.032), we recovered the sequence of the amphioxus RAG1 and RAG2 homologs. Unfortunately, it appears that *RAG1L* was not properly annotated in either of the two RefSeq genomes analyzed in this study, whereas *RAG2L* was only annotated in *B. belcheri* (with the gene ID LOC109474408). Although the annotation of both reference genomes, generated through the NCBI genome annotation pipeline, appears to be rather complete (just 2.6% and 1.3% metazoan BUSCO genes are missing/fragmented in *B. floridae* and *B. belcheri*, respectively), a small fraction of protein-coding genes may occasionally escape annotation, either due to lack of sufficient evidence, or due to technical issues (e.g., assembly errors, poorly resolved repetitive regions, etc.). Thus, unfortunately, we could only analyze the presence/absence patterns of *RAG2L* in *B. belcheri*. Our analysis showed that *RAG2L* is a core gene. Although we could not directly assess PAV for *B. floridae* RAG2L and for RAG1L in both species, the fact that their annotated flanking genes were, in all cases, part of the core gene set, suggests that amphioxus *RAG1L* and *RAG2L* genes are likely not dispensable. In addition, we performed a dedicated PAV analysis for the loci harboring the non-annotated RAG genes in *B. belcheri* and could show that the corresponding genomic regions are always part of the core. Due to the lack of direct

evidence regarding the PAV status of the RAG genes, we prefer not to mention these results in the main document.

3) What is not investigated in depth are details of the chromosomal locations where PAV occur. It would be interesting to see whether the sequences surrounding gene gain and loss show common features (e.g. transposon mediated integration would lead to target site duplication). Even if a detailed analysis would likely be beyond the scope of this manuscript, the authors could may be add a few sentences in the discussion about the mechanisms by which they envision these events to occur.

We would like to thank the reviewer for this suggestion, since the impact of transposable element activity has been previously suggested to be quite important in shaping the structure of pangenomes. To tackle this, we have added two new analyses to the manuscript. First, we added a new figure which summarizes the chromosomal distribution of dispensable genes in *B. floridae* (we did not generate a similar figure for *B. belcheri*, since the genome of this species does not reach a chromosome level). We briefly discussed the distribution of dispensable genes, highlighting the lack of statistically significant differences among chromosomes, even though some local regions with an increased density of dispensable genes were observed. In addition to this, we further implemented an analysis to indirectly estimate the size distribution of dispensable genomic regions, exploiting the concordant or discordant patterns of PAV between pair of dispensable genes encoded by the same chromosome or scaffold. This allowed us to test whether lower pairwise distances among dispensable gene pairs were associated with higher concordance values, as this would imply a pattern of shared inheritance due to the association with the same dispensable "block". This approach provided important support to our analyses, since we identified a correlation of PAV patterns higher than chance for pairwise distances <25Kb in *B. floridae* and <30Kb in *B. belcheri*.

Second, we exploited the species-specific repeat libraries generated for our analysis, which classified each element in different classes, allowing the determination of their genomic locations. Based on the positions of dispensable genes, we investigated, through hypergeometric tests, whether any of the detected types of repeats were significantly over-represented in the regions flanking each dispensable gene (or block of contiguous flanking genes). This allowed us to test the hypothesis that certain types of repeated elements could be associated with dispensable genes and thereby be tentatively implicated in being major drivers of structural rearrangements in amphioxus genomes. These results are now reported in a novel supplementary table. Overall, the evidence in support of this hypothesis was rather weak, as the over-representation of just a very few repeat types was supported by significant p-values, and in any case a minor fraction of dispensable genes was associated with such slightly over-represented repeats.
