# Supplementary material for: The highly dynamic pangenome of basal chordates is enriched in defence and immunity genes and is inherited following the Mendelian law
Source: PLoS Genet. 2025 Aug 18;21(8):e1011833. doi: 10.1371/journal.pgen.1011833 (PMC12373286; doi:10.1371/journal.pgen.1011833)
Supplement: Attachment — Submitted filename: response to reviewers_FINAL.pdf [file pgen.1011833.s017.pdf]

PGENETICS-D-25-00112R1 – Response-to-Reviewers

Dear *Plos Genetics* Editor and Reviewers,

We thank you for your efforts in evaluating our manuscript and for the suggestions on how to improve the comprehension of our analysis. We have carefully revised the text according to your comments, and hope that, in this revised form, our manuscript is suitable for publication in *Plos Genetics* as a research article. Below we provide our point-by-point responses.

Reviewer #1

The overall manuscript has improved greatly after the revision. The manuscript is now much more readable and the results and conclusions stated more logically. I appreciate the effort by the authors. As stated previously I find this work interesting, adequate and an important contribution to amphioxus and metazoan genomics. Having said this, I find several major and minor issues still need to be addressed.

We thank you for these additional comments. We have further modified our manuscript to address these points.

Major comments:

- I find the figures are still not always properly framed and interpreted in the results section. The results describe the overall outcome of each figure. However, they sometimes fail to fully explain the details that can be seen in the figure, and they frequently leave the reader to interpret and guess the consequences. I left comments on the most important cases as minor comments, but I would appreciate an overall effort to revert this tendency.

We have further considered the description of the figures, and added, when necessary, additional text to more comprehensively describe the figures' contents. As commented among the major and minor points, we have modified several panels (e.g., Fig1b, Fig3b-c-d, Fig6, Fig7 and Fig8) to make them more comprehensible, avoiding unnecessary complexity.

Is it expected that the inheritance of PAV would follow Mendelian patterns of inheritance? What would be the alternatives? Please develop on this in the introduction and discussion sections.

We have previously demonstrated that, in other biological systems, dispensable genes are inherited following Mendelian patterns (Gerdol et al. 2020), as would be expected in any biallelic locus (in this case, one allele represents gene presence and the other represents gene absence (a null allele). However, this expectation cannot be a priori expected to be universal, because dispensable genes are often embedded within large structural variants that may impair homologous chromosome pairing during meiosis. From a theoretical standpoint, such disruptions can lead to segregation distortion or non-random transmission, resulting in deviations from expected Mendelian ratios in progeny. Additionally, although each dispensable gene can be individually absent, the combined absence of multiple functionally redundant dispensable genes may not be equally tolerated. In such cases, selective constraints may limit the viable gene PAV configurations across individuals. Such concepts were developed in the Introduction and Discussion sections, as suggested by the reviewer.

The use of the term "pangenome" in some sections of the manuscript is still confusing. It is used as synonym to "dispensable part of the genome" and in some sentences it can still be interpreted that not all species have by definition a pangenome. This is misleading and in some cases, it can lead to misunderstandings. I point the most critical cases as minor comments, but I would appreciate it if the authors would carefully read all the text once more, being attentive to this.

We have revised the text to remove sentences where the term 'pangenome' was used in a misleading manner, to align with the definition provided in the Introduction (*i.e.,* pangenome as the sum of the genetic information of a given species, as detailed on lines 58-75).

I understand that the methods are very explicit and that the PAV analysis and GO/Pfam enrichment use already published pipelines with few modifications. However, I must insist on publication of the full code used in the study in order to make the research in this manuscript fully reproducible. The use of common R packages should not excuse authors from publishing the code that uses them. Commands executed on the data previously or between pipelines can affect the results significantly, and the code to generate the figures is very important for their reproducibility. The manuscript would benefit greatly if the code to reproduce the analysis and figures is made available via a public repository. This is currently the standard of good practice in bioinformatics.

We have uploaded the R script together with all the required files to run it to *github* (https://github.com/umberos/Lancelet_pangenome). This will ensure the reproducibility or future reuse of our methods. This is now stated in the M&M section (see lines 647-648).

Finally, a thorough re-reading with a critical eye would greatly improve the text's cohesion and clarity.

We have amended several points in the text.

Minor comments:

L106: B. lanceolatum also has a chromosome level assembly (see reference 65)

The reference is now also cited in the Introduction, when mentioning the chromosome-scale genome of *B. lanceolatum*.

L109: Please delete "presence of" since what this study investigates is not the presence of a pangenome but its architecture.

Removed.

L145: Please cite Table S1 for the reference of the transcriptomic datasets.

We now mention Table S1 (line 157).

T1: Change "No. of datasets" to "No. of samples" or "No. of individuals" which are more clear.

Modified.

F1b: The scale of the axes does not allow for the appreciation of the correlation. Please zoom in. Also, for B. belcheri there are two samples (presumably the two samples used to generate the reference genome assembly) that clearly cluster separately from the others and drive the correlation. Please, specify which samples they are to justify why they cluster apart. If they are the two reference samples, consider calculating the strength of the B. belcheri correlation without them, since they are a clear exception to the analysis.

We divided the two species into two different panels to use a proper axis scale for each of them. The two samples clustering apart are the two short-read archives used to build the reference genome. These samples, as expected, have a very limited number of absent genes and regions. This was already mentioned on lines 151-154 (now stated more clearly and have also been removed from Fig1b).

L164: B. floridae is not the only species with chromosome level assembly. See L106 and reference 65.

Thanks, we removed this part of the sentence since it was incorrect.

F3b: Please rephrase the caption and associated text in results and add a more clear explanation. It is not obvious to understand what the figure shows and means. Also, consider changing figure labels to indicate that they refer to "coverage" ratios.

Thanks for highlighting this aspect. We have decided to propose a different figure to depict coverage ratios. We have also slightly expanded the Discussion of this part (553-556).

F3c & d: In the results text, they are wrongly referenced (F3c must be F3d and vice versa).

Corrected.

F3c & d and related text: It is not clear what "SNP frequency" refers to. See, for example, the caption of the figure where "SNP frequency" is used to describe both the prevalence of given SNPs among samples (F3c y axis) and the distance between SNPs along the genome/virus (SNP every x nt in F3b y axis). Please clarify in the results text, figure caption and figure labels.

F3c: I don't understand what F3c is representing and what its message is. The results text says that all viral SNPs appear mostly at a frequency of 1 or 0.5 (across samples). How can SNP frequency across samples be plotted per sample? Please rephrase and clarify both in the results text and figure caption. Also, a boxplot is not appropriate to show such extreme distributions of frequencies. Please, find another way of representing it. Why do two samples look so different from the rest? Please, explain in the results text how to interpret the figure results.
F3d: What do the lines linking the points on one boxplot and the other represent?

We modified Fig3 to take into consideration all the suggestions and we hope that in the current form it is more readable. As shown in the revised panel, the two largely divergent samples display a very low number of SNPs (i.e. 4 and 6, respectively), detected with low frequency. In our interpretation, this most likely indicates that the integrated viral sequence in these two samples was virtually identical to the sequence found in the reference genome, whereas the few detected SNPs are likely artifacts due to sequencing errors.

T2 & T3: T3 is referenced before T2. Please, change labels accordingly.

Thanks, we corrected the numbering in the text.

L261: Please change "pangenomes" to "dispensable genes". What this section analyses are the potential functional consequences of the dispensable genes, not the entire pangenome which includes the whole genome.

Modified.

L265: Please develop further on the results. What type of Pfam domains are enriched in dispensable genes?

This section was expanded.

L267: Please change the sentence starting at this line to something like: "a and b. Volcano plot depicting the enriched Pfam domains encoded by the shell/cloud dispensable genes in B. belcheri (a) and (b) B. floridae". Same for the sentence starting at L272 referencing figures F4d & F4e and at L 279 for figures F4h & F4i (note the formatting in this last one).

Modified, as suggested.

L320: I don't see how this is the conclusion of such a paragraph. Please rephrase or if it is intended to be a conclusion of the entire section, please make it clear.

We removed this sentence.

F6b & F6c: Why are only reference sequences labeled as dispensable? Aren't the further sequences discovered while building the pangenome also dispensable by definition? Please clarify in the figure or text or explain if I'm getting something wrong. Same for F7b.

Since all the genes absent in the reference genome are dispensable by definition, we did not think that it is necessary to mark them as such. Since most of the sequences depicted in the trees are dispensable, we considered that additional asterisks would render the figure overcrowded. We therefore chose to switch this indication to core genes in the updated versions of panels 6b, 6c and 7b.

F6d: This figure needs a better contextualization and labeling to link it to the labels in figures b and c. Same for F7c.

We added labels to panels 6d and 7c to clarify the links between the sequence subgroups shown in the trees and domain organizations.

F8a-d: These are very methodological figures. They show no results. Please, move them to the supplementary materials.

We agree with this observation and, accordingly, redraw Figure 8, moving the technical panels to the new Figure S5.

L422: Please, cite F8e in the sentence that finishes here.

Added.

L435: Please rephrase. This sentence implies that not all species necessarily have a pangenome.

We modified this sentence and double-checked the text to remove these inaccuracies.

L544: Please discuss why you think that gene duplication and exon shuffling are the main cause of the generation of the dispensible part of the genome. Are your results suggesting something like this? Isn't virus genome integration another mechanism that your results suggest having had a role in it?

This sentence was rewritten to clarify the impact that gene duplication and viral integration may have on the generation of the dispensable part of the pangenome.

Reviewer #2

The manuscript has clearly improved from the previous version in terms of readability, and it will hopefully attract the broad readership of PLoS Genetics to a research area that has been largely ignored thus far by focussing on lab model organisms that exhibit rather limited genetic diveristy (if at all).

Thanks for the positive comments.
